# Driving Therapeutic Innovation in Neurodegenerative Disease With Hydrogen Deuterium eXchange Mass Spectrometry

**DOI:** 10.1016/j.mcpro.2025.101017

**Published:** 2025-06-20

**Authors:** Andrea Pierangelini, Benedikt M. Kessler, Darragh P. O’Brien

**Affiliations:** Target Discovery Institute, Centre for Medicines Discovery, Nuffield Department of Medicine, University of Oxford, Oxford, United Kingdom

**Keywords:** neurodegeneration, therapeutics, drug discovery, structural proteomics, HDX-MS

## Abstract

Human neurodegenerative conditions such as Parkinson’s and Alzheimer’s disease are characterized by the formation and deposition of toxic protein species which exacerbate neuronal dysfunction, impacting the structure and function of the healthy brain. Deciphering the mechanisms underlying protein (mis)folding and aggregation is not only essential for a more coherent view of neurodegeneration, but also crucial for the development of novel therapeutics targeting this family of disorders. Key pathological drivers of neurodegeneration, such as alpha-synuclein and tau proteins, have traditionally proved extremely challenging to characterize structurally due to their intrinsic and widespread structural plasticity. Hydrogen-Deuterium eXchange Mass Spectrometry has emerged as a powerful tool to help circumvent this, owing to its ability to capture protein intrinsic disorder in solution, in addition to the transient structural conformations that typify protein aggregation pathways. This review brings together the most recent research where Hydrogen-Deuterium eXchange Mass Spectrometry has shed light on mechanisms of neurodegeneration. We highlight how the technique has been successfully integrated into therapeutic development workflows targeting some of the most prevalent neurodegenerative diseases.

## Highlights

• Understanding the mechanisms underlying neurodegeneration enhances neurotherapeutics.
• HDX-MS deciphers the conformational dynamics of protein function and regulation.
• HDX-MS profiles therapeutic antibodies and drug-induced protein changes.

## Graphical Abstract

**Figure:**
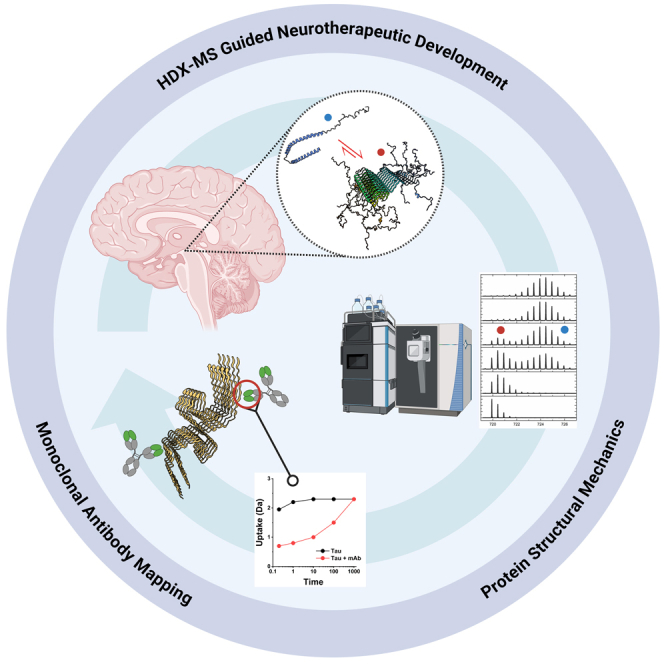

## In Brief

Deciphering the mechanisms underlying protein misfolding and aggregation is key to a more complete understanding of human neurodegenerative disease and the development of targeted neurotherapeutics thereof. HDX-MS provides a structural proteomics solution for monitoring protein conformational dynamics and the intrinsic and widespread structural plasticity associated with key drivers of neurodegenerative disease, including alpha-synuclein and tau proteins. The technique is being fully integrated into modern therapeutic regimes to aid design of inhibitors to limit pathological protein aggregation and for the development of neurotherapeutic mAbs.

Neurodegenerative disease encompasses a collection of disorders which display a progressive decline in the normal function and structure of the nervous system. Primarily centered on the brain and spinal cord, the gradual loss of healthy neurons and supportive glial cells can dramatically alter behavior and motor function, often leading to impaired cognition and memory, the culmination of which can significantly reduce both health and life span (1). The most prevalent and well-known human neurodegenerative diseases include Alzheimer’s (AD) and Parkinson’s diseases (PD), but less common forms such as amyotrophic lateral sclerosis (ALS), multiple sclerosis, and Huntington’s disease also exist. In addition to complex genetic components, many of these diseases are characterized by dysregulation at the protein level, including the development of toxic protein aggregates which can detrimentally impact brain physiology (2, 3, 4). Proteomics, the large-scale study of proteins in biological systems, lends itself perfectly to the study of such protein dysregulation. Recent advances in the technology have significantly advanced the understanding of neurodegenerative disease mechanisms, the development of novel drugs and therapeutics toward the pursuit of personalized medicine, and finally, in biomarker discovery, where proteomics has aided in the identification of protein species that can be used for early diagnosis, tracking disease progression, and evaluating treatment response (5, 6, 7).

Protein structural plasticity is a key hallmark of several proteins involved in neurodegenerative disease, including alpha-synuclein in PD, tau in AD, and TAR DNA-binding protein 43 (TDP-43) in ALS (8). Physiologically, these proteins are involved in a diverse range of cellular functions including the regulation of neurotransmitter release (9), microtubule stabilization and neuronal support (10), and RNA splicing, transport, and stability (11). Pathologically, they can be heavily modified posttranslationally and undergo an aggregation cascade of protein misfolding, organization, and accumulation, ranging from a soluble state of small monomeric or oligomeric species, to an insoluble fibrillized state typified by higher order fibrils and proteinaceous deposits (12). Whether insoluble protein aggregates are the causative agent of neuronal dysfunction is currently subject to fierce debate; rather, soluble amyloid and oligomer intermediates have been implicated as being the most toxic variants (13). Limiting protein misfolding and aggregation, or abolishing it completely, is therefore one of the key goals in the development of novel therapeutic interventions across the spectrum of neurodegenerative disease. Such interventions can include chemically derived small-molecule enzyme inhibitors, activators or enhancers, peptides, natural products, or more recently, disease-modifying biologics such as MAbs that can help clear protein aggregates through the host immune response (14, 15, 16, 17). Understanding these systems from a molecular perspective is key to the development and success of these products, but protein structure-function relationships have traditionally been technically very challenging to address experimentally, due to the inherent structural disorder and widespread modification of proteins of interest. Mass spectrometry (MS) and structural proteomics may hold the key to unlocking some of these secrets.

## HDX-MS as a Structural Proteomics Tool to Monitor Protein Conformational Dynamics

**Fig. 1:**
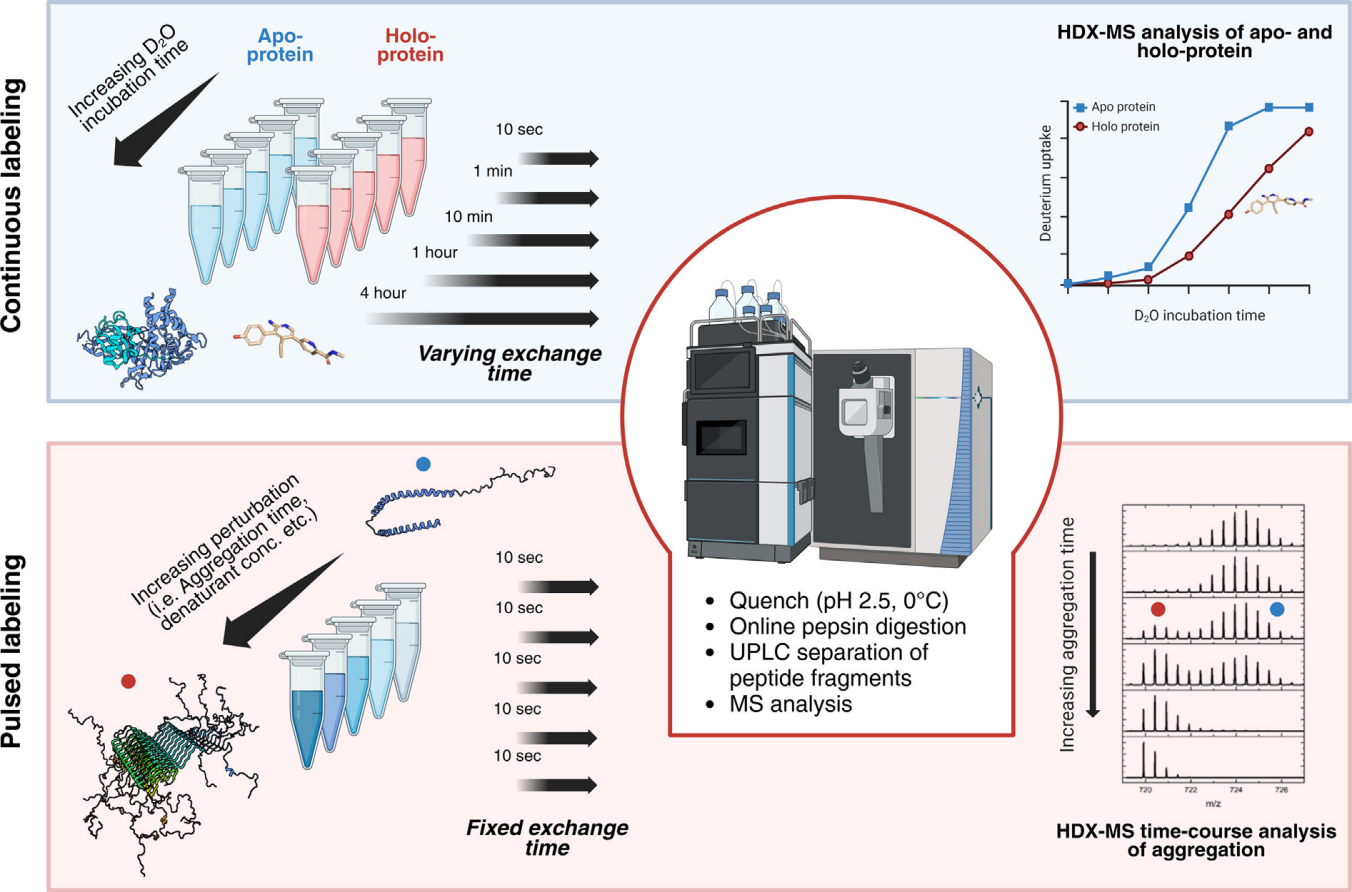
**Continuous and pulsed labeling HDX-MS workflows to monitor neurodegenerative mechanisms**. In a continuous labeling experiment (*upper panel*), proteins (*i*.*e*., apoprotein *versus* holo-protein, WT *versus* mutants) are incubated in deuterated buffer for varying lengths of time to monitor structural changes over time. In contrast, pulsed labeling (*lower panel*) involves incubating a single protein of interest under different condition (*i*.*e*., increasing aggregation time or rising denaturant concentrations). Induced structural changes are probed by a short pulse of deuterated buffer, typically 10 s. In both approaches, labeling is followed by quenching at acidic pH, on-line protein digestion, and separation of the resulting peptides *via* UPLC prior to MS analysis. These two approaches address different biological questions in a complementary manner, as is the case of aggregation; continuous labeling can provide insights into how small-molecule drugs impact aggregation mechanisms, whereas pulsed labeling assists in deciphering the kinetics of aggregation itself. Figures were prepared with the help of BioRender Software. HDX-MS, Hydrogen-Deuterium eXchange Mass Spectrometry.

A comprehensive understanding of the intricate molecular mechanisms that drive neurodegeneration is essential for developing effective treatment strategies. Investigating protein structural dynamics and protein interactions with physiological partners is crucial to better understanding and treating disease (8, 18). Despite significant recent advances in structural biology, several gaps remain in our understanding of the dynamic processes that contribute to the pathological changes observed in neuropathy. Over the past two decades, Hydrogen-Deuterium eXchange Mass Spectrometry (HDX-MS) has emerged as an extremely powerful tool to help fill in some of these gaps, owing to its inherent ability to measure protein structural disorder, while capturing the transient structural conformations that characterize proteins in solution and which are mostly inaccessible to static techniques such as X-ray crystallography (19). HDX-MS is based on the relatively straightforward concept that backbone amide hydrogens exchange with deuterons when exposed to deuterium oxide, resulting in a mass increase that can be easily measured in a mass spectrometer (20, 21). Isotopic exchange is influenced by several factors including pH, temperature, ionic strength, and the structure and dynamics of proteins. Insights into protein dynamics are provided by the dependence of the exchange rate on the solvent accessibility and local hydrogen bonding environment of protons at different sites. Protons located in solvent-exposed regions exchange much more rapidly compared to those that are engaged in H-bonding or that are buried within the protein’s core (22). This happens because the secondary structure housing these protons must transition from a “closed” conformation to an “open” one where the H-bonding network is temporarily disrupted and the amide protons become exchangeable (22). The kinetics of this conformational switching can be described with two rate constants: the opening rate constant (*k*_op_) and the closing rate constant (*k*_cl_) (Equation 1):

(Eq. 1) $$N-H_{closed}\;\begin{array}{c} k_{cl}\\ ⇋\\ k_{op} \end{array}\;N-H_{open}\begin{array}{c} ↔\\ k_{ch} \end{array}\;N-D_{open}\;\begin{array}{c} k_{cl}\\ ⇋\\ k_{op} \end{array}\;N-D_{closed}$$

where *k*_ch_ defines the chemical exchange rate. HDX primarily occurs in the open state and depending on the relative rates between *k*_ch_ and *k*_cl_, two different exchange regimes can be distinguished: *EX1 exchange*, where the closing event is slower than the chemical exchange. As a result, all hydrogens in a region exchange during a single opening event. This mechanism is typically observed in systems undergoing unfolding or conformational changes, such as with amyloid fibrils or proteins exposed to denaturing agents (Fig. 1) (23, 24, 25). *EX2 exchange* is the regime typically observed under physiological conditions. Here, the closing event occurs rapidly, and each amide hydrogen exchanges independently based on local dynamics and solvent accessibility.

Experimentally, a standard HDX-MS workflow is divided into several established steps (Fig. 1) (26); proteins are first exposed to deuterium oxide over a time-course of seconds to hours to facilitate hydrogen-deuterium exchange. Labeling conditions can be refined by adjusting pH, temperature, and by adding cofactors or ligands. The exchange reaction is quenched by rapidly reducing the pH to ∼2.5 and the temperature to 0 °C. This step is crucial for minimizing the back-exchange of deuterium to hydrogen and preserving the incorporated deuteration information. To enhance downstream protein digestion, denaturing agents such as guanidinium and reducing agents such as tris(2-carboxyethyl)phosphine may be used to reduce disulfide bridges and facilitate better unfolding of the protein. Upon quenching, proteins are typically digested on-line with an immobilized acid-stable protease such as pepsin, generating overlapping peptides and providing resolution of HDX behaviors to within a few amino acid residues. Peptides are then separated *via* liquid chromatography and identified and analyzed by tandem MS. As the chromatographic separation is performed in aqueous buffers, the system is kept at 0 °C and pH 2.5 to minimize back exchange, and the separation of peptides is kept as short as possible. Deuterium uptake data are then extracted and mapped onto the protein sequence. Analysis involves comparing uptake patterns under different conditions or time points to infer structural and dynamic changes. Computational tools such as HDExaminer, DynamX, and MEMHDX can facilitate the processing, visualization, and statistical analysis of the data (27, 28). The accuracy of data analysis is enhanced by integrating control experiments to account for factors such as back exchange.

Depending on your question of interest, two different HDX-MS regimes are useful: Continuous labeling, where proteins are exposed to deuterated solvent for varying incubation times, allowing for the progressive exchange of amide hydrogens. This method, which is the most widely implemented, is particularly useful for studying the overall dynamics of a protein, the effect of mutations on its flexibility, or to follow binding events and the structural perturbations that may follow (Fig. 1) (29). Pulsed labeling on the other hand is ideal for studying dynamic processes such as protein folding, aggregation, or stressor-induced conformational shifts (30, 31). The protein under investigation is first exposed to external stimuli such as denaturants, temperature, or aggregation-inducing factors, and only then is it briefly exposed to deuterated solvent for a set period. In this way, it is possible to capture transient intermediates or rapid conformational changes, such as those typical of alpha-synuclein or amyloid aggregation (19).

## HDX-MS to Guide Drug Discovery and Design

**Fig. 2:**
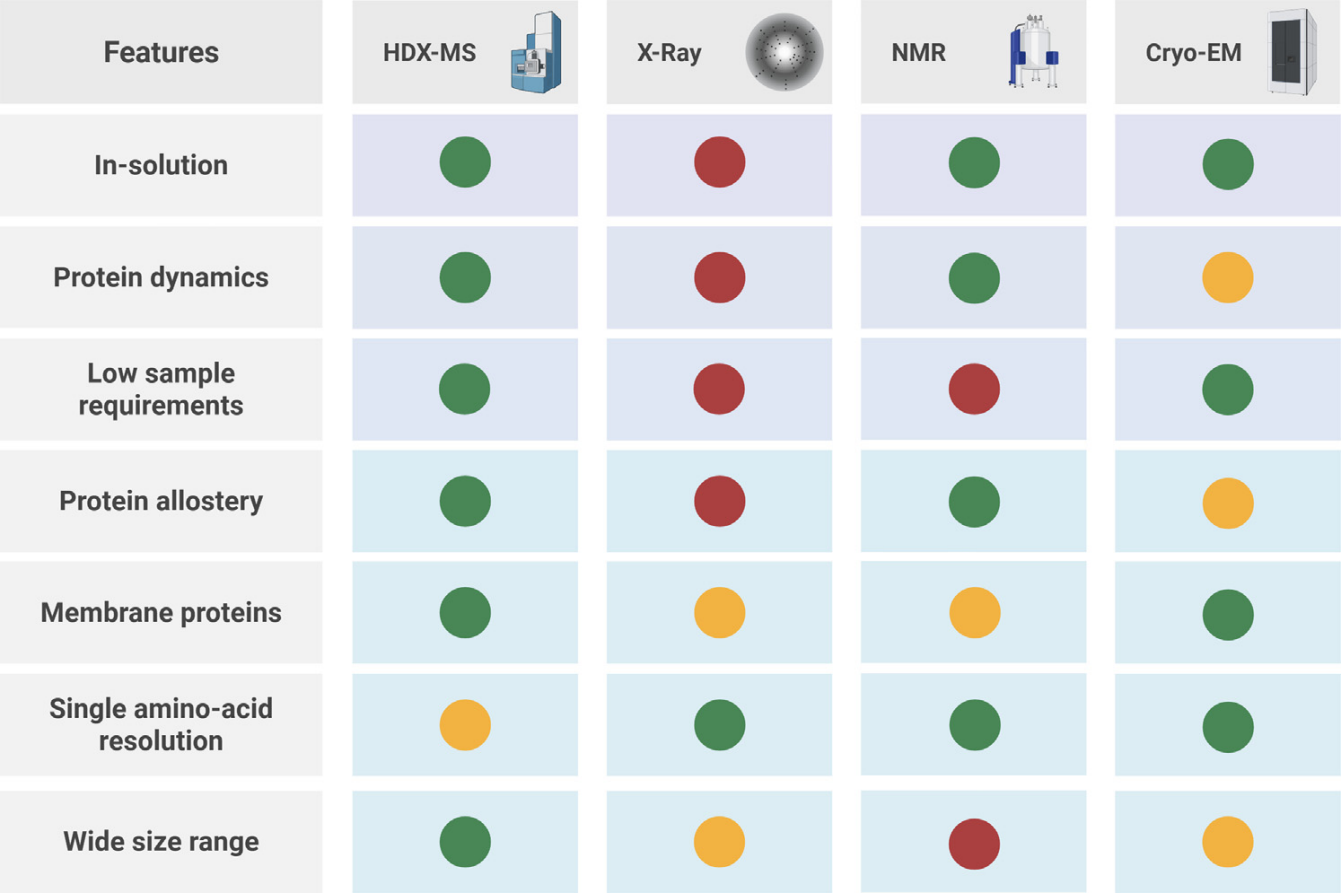
**Comparative overview of structural biology techniques to investigate protein structure and dynamics**. HDX-MS has several advantages over other structural approaches in terms of providing information on protein dynamics and allostery in an in solution, native environment. This can also be achieved in challenging systems, such as those that observed with the addition of membrane proteins. Both large and small proteins are feasible. The technique consumes low sample amounts, but is limited in its ability to provide only medium resolution information. Generally speaking, HDX behaviors can only be localized to short stretches of peptide sequences, rather than to within a single amino acid. A traffic light system has been used to ease comparison to other techniques, where a *green circle* represents good applicability of the technique, *red*, poor applicability, and *amber* meaning there may be challenges encountered. Figures were prepared with the help of BioRender Software. HDX-MS, Hydrogen-Deuterium eXchange Mass Spectrometry.

When studying the interaction between proteins and other molecules in a native, physiological-like environment, HDX-MS offers several key advantages over other structural methods (Fig. 2). Although it is undeniable that X-ray crystallography still represents the gold standard for elucidating interactions between drugs candidates and biological targets, this technique only provides a static snapshot of protein structure, offering limited insights into protein dynamics (32). Monitoring protein movement is essential when studying neurodegenerative disease, as accumulating evidence indicates that abnormal and pliable protein conformers play a significant role in disease pathogenesis (33). The application of X-ray crystallography in drug development for neurodegenerative diseases is further complicated by the fact that many proteins involved in these pathologies are often physiologically unstructured (34, 35), as is the case for alpha-synuclein in PD and tau in AD (36, 37). Furthermore, the transition of these proteins from monomers to toxic oligomeric species occurs through transient and heterogeneous intermediates, which are extremely challenging to capture experimentally (33, 38, 39). HDX-MS can overcome these technical barriers, providing residue-level, in-solution insights into the conformational dynamics underlying protein function in a native environment (40), as well as insights into allosteric regulation information that are important for developing novel pharmaceutics (Fig. 2) (41, 42, 43). Such dynamics are often driven by ligand binding or protein interaction, which may alter the overall structure of target proteins, thereby modulating their function (44, 45). This structural regulation impacts protein function in various ways, including stabilization or destabilization of functional domains, allosteric regulation of activity, and blocking or enhancing protein–protein interactions (PPIs) (46, 47, 48, 49). As proteins are typically digested into small peptide fragments in HDX-MS, the size of the protein therefore becomes redundant, as long as there is sufficient resolving power in the downstream liquid chromatography separation of peptides. Size constraints are an issue in analogous structural tools; high-resolution cryo-EM is challenging for proteins that are very small, and conversely, it is difficult to obtain highly resolved X-ray crystal structures of very large protein complexes (Fig. 2). Given these advantages, HDX-MS has therefore become an invaluable tool for studying a wide range of molecular interactions in neurotherapeutic development regimes. In the following section, we provide a brief overview of its applications in characterizing protein–small molecule interactions, PPIs, and functional regulation by posttranslational modifications (PTMs), while referring the reader to more comprehensive reviews for more in-depth discussions on each individual topic (50).

### Small-Molecule Drugs

One of the most widespread applications of HDX-MS is to characterize small-molecule binding to proteins to decipher interaction interfaces and the mechanisms of action of drugs. It requires relatively little material, allows the analysis of low dissociation constant interactions, and can accommodate complex biological environments like lipids, without detrimentally affecting data quality (Fig. 2) (50, 51, 52, 53). It is particularly useful for investigating how ligand binding affects protein dynamics, as crystallography alone is not always able to detect subtle conformational changes (54). In neurodegenerative disease, understanding how small molecules interact with proteins and interfere, for example, with aggregation cascades, is crucial for developing new therapeutic interventions. A recent example of this explored the impact of the small molecule resveratrol on alpha-synuclein aggregation in PD (25). Through integration of HDX-MS with other techniques, it was shown how resveratrol destabilized key regions of the protein, leading to alternative protein aggregation and reduced fibril formation. HDX-MS has also proven to be invaluable in classifying drug action, as its ability to distinguish between agonist, antagonists, and partial agonist, has been reported. While traditional structural methods often struggle to identify ligand-induced conformational shifts (55), a recent study on the β1-adrenergic receptor demonstrated how HDX-MS could distinguish ligand classes through their effect on a critical loop, thereby enabling a rapid stratification between agonist and antagonist molecules (56). There are now numerous studies reporting the applicability of HDX-MS for characterizing small-molecule binders and allosteric regulation of proteins, across a plethora of human disease areas (49, 57, 58, 59, 60, 61, 62).

**Fig. 3:**
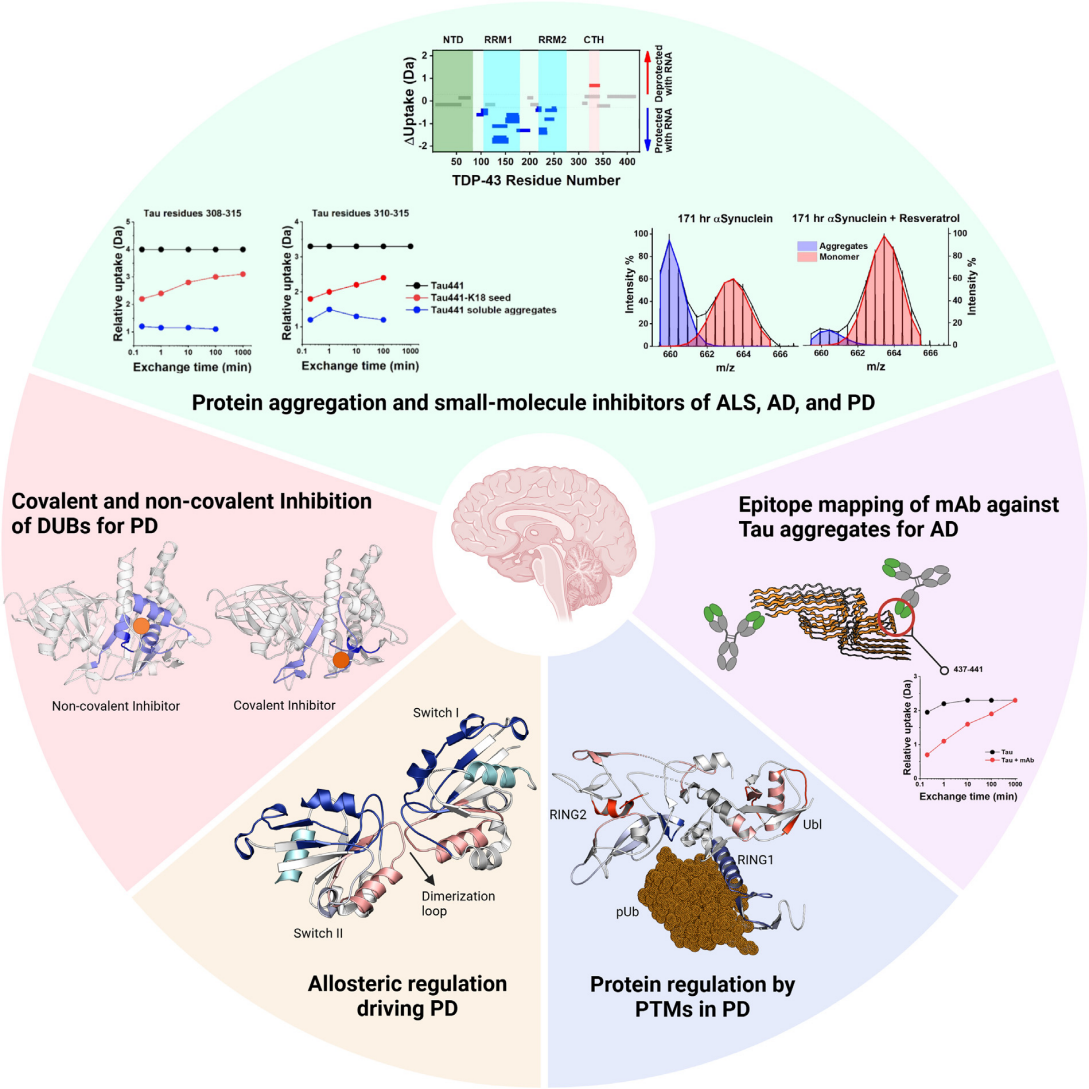
**HDX-MS across the field of neurodegenerative disease research**. *Clockwise from top*, *green panel*: HDX-MS in investigating protein aggregation mechanisms and small-molecule inhibitors in AD (*left*), ALS (*middle*), and PD (*right*) (adapted from (25, 222)). Huang *et al*. employed HDX-MS to study the structural plasticity of monomeric tau and of its soluble aggregates. Minshull *et al*. investigated the effect of RNA binding on TDP-43 aggregation propensity. By employing pulsed labeling HDX-MS, Illes-Toth and colleagues monitored the effect of resveratrol on alpha-synuclein aggregation. *Purple panel*: Huang *et al*. also demonstrated how HDX-MS can be used for deciphering the binding epitope of antibodies directed against tau aggregates (adapted from (76)). *Blue panel*: Gladkova *et al*. employed HDX-MS to study the mechanism of Parkin activation following phosphorylation by PINK1, revealing important domain rearrangement during the activation process (adapted from (103)). *Orange panel*: Deyaert *et al*. discovered interdomain allosteric communication in Roco proteins, providing insights into possible mechanisms underlying LRRK2 role in PD (adapted from (155)). *Red panel*: our group employed HDX-MS to study the distinct structural mechanisms by which covalent and noncovalent inhibitors interact with USP30, a mitophagy-regulating enzyme involved in PD (adapted from (63, 64)). Figures were prepared with the help of BioRender Software. HDX-MS, Hydrogen-Deuterium eXchange Mass Spectrometry; LRRK2, leucine-rich repeat kinase 2; PD, Parkinson’s disease; TDP-43, TAR DNA-binding protein 43; AD, Alzheimer’s disease; ALS, amyotrophic lateral sclerosis.

A very recent application of HDX-MS in driving neurodegenerative disease drug design comes from our own lab (63, 64). We first investigated the structural dynamics of USP30, a mitochondrial deubiquitinating enzyme (DUB), in complex with a noncovalent benzosulfonamide inhibitor, **USP30_inh_**. The importance of targeting USP30 lies in its critical role in regulating mitophagy (65). Evidence suggests that loss of opposing E3 ligase parkin activity is pathogenic in both sporadic and inherited forms of PD, and loss-of-function mutations in its gene results in a rare form of autosomal recessive PD (66). Inhibiting USP30 has therefore been proposed as a strategy to enhance mitophagy and protect neurons from degeneration (67). To elucidate the inhibitory mechanism of **USP30_inh_**, we integrated multiple biophysical and biochemical techniques, including activity-based protein profiling MS, enzyme kinetics, HDX-MS, and molecular docking simulations (63). Our study was the first of its kind to provide detailed structural and mechanistic information on the interaction of USP30 with an active small-molecule drug. The utility of HDX-MS in addressing a previously unanswered question highlights its potential as a key technique for understanding protein conformational changes upon ligand binding, reinforcing its crucial role in modern drug development pipelines. We subsequently expanded on the study by comparing our results to those obtained on a covalent USP30 inhibitor, **USP30-I-1** (64). HDX-MS offered unique structural insights into the different inhibitory mechanisms of the two molecules (Fig. 3). When the covalent inhibitor binds to USP30, structural changes are mainly localized in the catalytic region, whereas much larger structural perturbations were observed for the noncovalent inhibitor binding. This observation suggests that the covalent inhibitor engages in a more targeted interaction, while the noncovalent inhibitor likely undergoes reorientation within the binding pocket (63). Ultimately, understanding how different classes of inhibitors interact with USP30 can guide the design of next-generation drugs with more tailored mechanisms of action.

### PPI and Protein–Membrane Interaction, including Antibody Epitope Mapping

PPIs are fundamental to nearly all cellular processes (18), including signal transduction, enzyme regulation, and protein aggregation. It is now known that under certain conditions, several proteins can interact with alpha-synuclein, thus modulating its aggregation, for example (68). HDX-MS may provide the perfect structural tool for characterizing such structural interplay (48, 69). Despite their prevalence throughout biology, comprehensive monitoring of PPIs is still seen as technically challenging (50, 70). Perhaps the most studied PPIs are those involving antibodies, as epitope mapping is critical for the successful development and characterization of therapeutic antibodies and vaccine development (71, 72). There are numerous examples of the successful use of HDX-MS for identifying binding epitope for antibodies (73), including the optimization of neutralizing antibodies as antivirals (74, 75).

In AD, a number of anti-tau aggregate antibodies have been mapped by HDX-MS, also to assess alloantibody pathogenicity, for example (76, 77, 78, 79, 80, 81, 82). In addition to identifying binding hotspots, HDX-MS can also provide valuable insights into structural rearrangements induced by antibody interaction (Fig. 3) (83, 84), or enable the identification of binding hotspots directly in patient serum (85). For years, the use of mAbs for treating neurodegenerative diseases was limited, as their passage through the blood-brain barrier is extremely challenging, notwithstanding the vast complexities of brain physiology (86). However, the interest in immunotherapeutic approaches for treating neurodegenerative disease was very recently renewed following the approval of several anti–amyloid-beta (Aβ) antibodies showing a modest benefit for the treatment of AD (87, 88, 89, 90).

### Mechanistic Insights into Protein Function and Regulation by PTMs

Protein dynamics are frequently regulated through allosteric mechanisms and PTMs. A better understanding of these phenomena using the information provided by HDX-MS is crucial when considering drug design, as they can significantly alter protein function, conformation, and their interaction with biological partners (Fig. 3) (91, 92). Glycosylation is one such PTM which assists in protein folding and stability, in addition to protein trafficking, cell receptor activation, immune system recognition and signaling, and the protection of proteins from degradation upon exposure to harsh environments (93, 94, 95). Glycosylation is particularly important for the efficient activity and recognition of antibodies, and as such, many HDX-MS studies and workflows have been developed for the measurement of glycoproteins to enhance antibody and vaccine design (71, 96, 97, 98, 99, 100, 101). PTMs are particularly relevant to protein quality control and clearance, the crucial role that the ubiquitin-proteasome system plays in protein degradation and the removal of defective or misfolded proteins which can impair neuronal function (102).

PINK1, a serine-threonine kinase, and Parkin, an E3 ubiquitin ligase, are essential enzymes involved in mitochondrial quality control. Both proteins play a vital role in the mitophagy and removal of damaged mitochondria (103). As described above, loss-of-function mutations in the Parkin gene are responsible for a rare hereditary form of early on-set Parkinsonism, resulting in the progressive build-up of toxic mitochondrial species (92). Molecular glues to help circumvent this have recently been characterized by HDX-MS (104). Furthermore, clearly identifying how phosphorylation drives Parkin activation and PINK1 activity is of absolute interest for developing novel treatments of the condition. HDX-MS has been employed by several groups to address both PINK1 autophosphorylation and the subsequent phosphorylation-induced activation of Parkin (103, 104, 105, 106, 107). HDX-MS supported the finding that autophosphorylation of PINK1 is needed for ubiquitin recognition and binding and causes structural rearrangements near its active site (108). HDX-MS was also used to investigate how mutations in the uncrystallized region of PINK1 affect the dynamics of the protein, revealing how these mutations lead to a failure in stabilizing PINK1 near the mitochondrial membrane, thus impairing substrate phosphorylation (106). HDX-MS has shown that phosphorylation of Parkin’s ubiquitin-like domain triggers conformational changes that release the enzyme from its autoinhibited state, exposing the RING2 catalytic domain necessary for the hyper-ubiquitination of mitochondria (103). These findings provide unprecedented insights into the structural changes that occur within the catalytic domain of the protein, as this domain is poorly crystallized due to its high structural flexibility.

### Current Limitations of HDX-MS

As outlined above, HDX-MS is an extremely powerful tool in drug discovery and development to help define interactions between drugs and their protein targets. In saying this, the technique has several limitations which are important to mention and warrant discussion.

The vast majority of current HDX-MS studies typically use highly purified recombinant proteins in an effort to achieve acceptable levels of data quality. The approach is therefore limited to those species which can be produced in sufficient quantities and to acceptable quality. Purified proteins then need to behave well in a highly controlled experimental system, which may not reflect how they behave endogenously. In the bottom-up HDX-MS approach, these recombinant proteins are then digested into peptide fragments. In theory, this means that even large protein complexes can be measured. In practice, this is a very different matter. The generation of complex spectra and multiple overlapping peptides can make data interpretation and accuracy very difficult. Furthermore, it is challenging to routinely provide information at single amino acid resolution. Labeling information is typically at the peptide level, making it difficult to pinpoint the exact residues involved in small-molecule binding, for example. Adding to this, the technique is reliant on complete and efficient proteolytic digestion, which can be tricky for hydrophobic, highly structured, and membrane-bound regions of a protein. The technique monitors changes in both the hydrogen bonding and solvent accessibility when exposed to a perturbant or a ligand. It can be sometimes difficult to readily decipher between these two phenomena. Care must be taken to limit back exchange, which can ultimately result in an underestimation of the amount of deuterium incorporated into a protein. This necessitates the need for highly specialized and expensive pieces of kit to control for speed, temperature, and accuracy in measurement, and highly experienced users are essential to maintain instrument performance. Using standard workflows, very rapid protein dynamics are usually missed. It is extremely difficult to measure very fast–exchanging hydrogens without specialized apparatus which can measure dynamics at the millisecond timescale. Taking all of this into account, however, one of the greatest hurdles in the HDX-MS field still remains that of data handling and analysis. While the experiment itself can be relatively straightforward in theory, the comparison of multiple peptides of multiple proteins, across multiple states can lead to very large datasets. Then layering on multiple peptide charge states, multiple labeling time points, and multiple replicates, the size and complexity of data analysis can very quickly escalate.

These limitations have been readily identified and the HDX-MS community has been working hard to provide solutions. Very recent studies have captured protein dynamics of endogenous proteins from highly complex matrices by immunoprecipitating ultralarge protein targets from mammalian cell lines prior to the labeling experiment (109). Alternative gas-phase peptide MS fragmentation approaches such as electron transfer dissociation and electron capture dissociation are now available which can provide labeling information at the single residue level (110, 111). Further improving on the spatial resolution of the approach, alterative acid proteases to the well-established pepsin enzyme have now been identified, including protease XIII and nepenthesin I and II (112, 113, 114). These can be used either individually or in a multiprotease fashion HDX-MS instrumentation is constantly being improved upon, with more methods to enhance both the speed and accuracy of measurement, whilst minimizing back exchange (115, 116, 117). Finally, novel software solutions are constantly being provided to help aid in data visualization and interpretation (28, 118, 119, 120). A very recent example of this has been the launch of a novel software from the Schreiner Lab to facilitate the automated processing and analysis of highly complex data-independent acquisition workflows from challenging biological systems (109).

## HDX-MS Applications in Neurodegenerative Disease

### Parkinson’s Disease

PD is a largely sporadic and multifactorial neurodegenerative disease resulting in problems with both motor and nonmotor function (121). Impaired motor function refers to a collective Parkinsonism which includes an archetypical tremor, muscle rigidity, and bradykinesia. As the disease advances, nonmotor symptoms become more apparent, typically entailing a progressive worsening of cognition, behavioral changes, and psychosis (122). The pathomechanisms underlying PD are still poorly understood, but a key hallmark of the condition is the abnormal intracellular assembly and aggregation of alpha-synuclein protein into Lewy bodies and Lewy neurites of affected brain regions, leading to neuronal degeneration. This predominantly occurs in the *substantia nigra pars compacta* of the midbrain, which controls voluntary motor control through the dopaminergic system (123). As such, the drug *Levodopa* works by replenishing the diminished dopamine levels observed in PD in this region and is the most effective therapy for PD currently available, albeit with several issues (124). Alpha-synuclein is therefore an excellent starting point for the development of more modern neurotherapeutics against PD, and as such, several HDX-MS studies characterizing its conformational dynamics have already been described.

#### Alpha-Synuclein

Alpha-synuclein is a monomeric, negatively charged intrinsically disordered protein of 140 amino acids in length, whose precise molecular function is still unclear (125). The protein is organized in a modular fashion comprising an amphipathic N-terminal segment spanning residues 1 to 60, a hydrophobic non-Aβ component (NAC) region covering residues 61 to 95 which is essential for protein aggregation, and finally, the acidic and proline-rich C-terminal domain, spanning residues 96 to 140, which lacks the tendency to form structure, but rather, may convey essential functional elements through PTMs (126, 127). Alpha-synuclein tends to be natively disordered in solution, although this is currently a hot topic of debate, and can become partially folded and helical in nature with increasing hydrophobicity, decreasing pH, or when exposed to a detergent or lipid-rich membrane environment (125, 128, 129, 130, 131, 132). The completely open, structurally disordered and flexible nature of alpha-synuclein has been confirmed by several continuous and pulsed labeling HDX-MS studies, where maximal deuteration of its exchangeable hydrogens occurs in as little as 15 s (125, 133, 134).

As the reputed key pathogenic species in PD, several studies have used HDX-MS to monitor the self-assembly, oligomerization, and misfolding of alpha-synuclein monomers into higher-order neurotoxic soluble oligomers and their intermediates (133, 134, 135, 136, 137, 138). This structural transition underlies a toxic gain-of-function mechanism, a better understanding of which could provide an attractive target for therapeutic intervention in PD and synucleinopathies on the whole (139). A major analytical challenge to overcome, however, is the transient nature of the oligomers themselves, and the high degree of structural heterogeneity that can manifest (38). HDX-MS may provide a solution to this, with its ability to resolve heterogeneous populations (134). The aggregation kinetics of alpha-synuclein is slow, and consequently, groups have generally preferred to use pulsed labeling experiments to monitor its structural transitions. The Gross group at Washington University in St. Louis has performed a large body of work on alpha-synuclein by pulsed labeling HDX-MS (133). They have found that the time-dependent aggregation of the protein is primarily driven by the hydrophobic NAC region, which is significantly solvent protected. Moreover, the group were able to identify two populations of distinct exchange kinetics in the NAC, which appear to be a key feature of the self-assembly of amyloidogenic proteins. The findings were supported by transmission electron microscopy and CD. The flanking N- and C-terminal regions did not become substantially protected or undergo significant conformational rearrangement, suggesting that they are not essential for misfolding. The same group used their pulsed labeling HDX-MS set-up, in addition to limited proteolysis, to explore the effect of the anti-amyloid polyphenol resveratrol (*trans*-3,5,4-trihydroxy-trans-stilbene) on alpha-synuclein aggregation (25). As introduced earlier, the compound destabilized and remodeled the N-terminal NAC regions of the protein, leading to a more open and disaggregated oligomer conformation during the aggregation cascade.

A second group which has performed extensive HDX-MS characterization of the structural dynamics underlying the aggregation of soluble alpha-synuclein oligomers is the Jorgensen group in Denmark. They have confirmed the presence of a protected and stable NAC region and highly disordered and flexible C-terminal tail of the protein (134). Moreover, the authors identified several novel regions within the membrane-binding N-terminal portion of the protein which are strongly protected against isotopic exchange in the oligomeric state. This region may also contain structural elements and could thus prove useful when targeting oligomer-specific conformations.

PD has a distinct genetic component (122). Several point mutations in the alpha-synuclein protein have been proposed to increase its rate of aggregation and alter dopamine homeostasis. These include the early-onset PD mutations at A30P, E46K, and A53T, and the late-onset PD variant H50Q (130). The structural impact of these mutations in driving pathogenesis has yet to be deciphered, but a couple of studies have used HDX-MS to better define this. The first study compared oligomers from WT alpha-synuclein to the three early-onset PD mutations listed above (137). Two distinct noninterconverting oligomeric species (types I and II) were observed across all variants, with type I able to dissociate into monomeric species by EX1 exchange kinetics. This ability is lacking in type II oligomers. Both forms are strongly solvent protected in the NAC region. Using a combination of global HDX-MS and native MS, the dose-dependent reduction in amyloid fibril formation of the A53T protein variant by two dopamine-derived catechols, 3,4-dihydroxyphenylacetic acid (DOPAC) and 3,4-dihydroxyphenylethanol has very recently been demonstrated (130). A less protected and open conformation of the monomeric protein in the presence of the catechols contributes to this reduced propensity for aggregation. To further understand the effect of DOPAC on synuclein function, Rizzotto and colleagues recently employed HDX-MS to investigate how this small molecule modulates the interaction of alpha-synuclein and its E46K mutant with biological membranes (140). The authors note the differing influences that DOPAC has on the binding dynamics of both protein forms to synthetic vesicles. HDX-MS revealed that E46K is more dynamic than WT alpha-synuclein when both are bound to membranes. Preincubation with DOPAC before addition of vesicles did not lead to further exposure in the E46K mutant but increased the flexibility of the bound WT alpha-synuclein. On the other hand, the incubation of DOPAC with the membrane-bound proteins had the opposite effect, leading to significant structural changes in the E46K mutant, but no effect on the WT protein. These findings suggest that the E46K structure is more solvent-exposed when bound to biological membranes compared to its WT counterpart, thus enhancing its accessibility to small molecules.

A recent study by Jorgensen and colleagues used HDX-MS to explore the ability of the small green tea antioxidant epigallocatechin gallate (EGCG) in reducing alpha-synuclein oligomeric cytotoxicity by preventing their interaction with, and permeabilization of, cell membranes (126). The authors highlight the coexistence of two oligomers in solution, OI and OII, and used HDX-MS to track the disassembly of multimeric OI into OII at the N-terminal domain of the protein. Oligomers which were exposed to EGCG underwent oxidation at key methionine residues, with significant structural and functional consequences at the N terminus; HDX-MS demonstrated a clear increase in the stability of folded structures at the N-terminal domain in the presence of EGCG, the nature of which may reduce the affinity of oligomer–membrane interactions, making them less cytotoxic (126). An analogous study used HDX-MS to monitor the structural transitions and exposure of the N-terminus of alpha-synuclein upon calcium binding to its C-terminal half, resulting in the protein having a higher propensity to aggregate (141), a phenomenon that could be targeted in the development of aggregation inhibitors.

Several groups have employed millisecond HDX-MS to trap and determine very quick exchange kinetics which can be lost when using longer labeling time points (131, 142). Wilson and colleagues employed time-resolved electrospray ionization HDX-MS to capture the interaction of alpha-synuclein with synthetic nanodisc membrane models comprising different headgroup charges, including zwitterionic DMPC and negative POPG (131). As expected, the DMPC membranes had no effect on the HDX-MS exchange behaviors of alpha-synuclein, whereas binding to amyloidogenic POPG induced substantial conformational changes within residues 19 to 28 and 45 to 57 of the protein, which provides further evidence of the “broken-helix” model of alpha-synuclein and membrane structural interplay, which ultimately drives aggregation. JJ Phillips and his team used custom-built hardware and software to determine the self-assembly and aggregation kinetics of monomeric, full-length alpha-synuclein on the millisecond timescale and at single-residue resolution (138). The novelty of this study was the introduction of buffer conditions that mimic the environment of extracellular, intracellular, and lysosomal cellular compartments, and the requisite application of an empirical correction to normalize HDX-MS data very acutely different solution conditions. The work highlighted a key region of the protein C-terminus that is crucial for its nucleation and aggregation, in addition to the opposing N-terminus and NAC regions, which drive fibril elongation. As an extension of this work, the authors characterized the effect of differing storage and chromatographic buffer conditions on the structural stability of high-molecular weight alpha-synuclein species by extensive biochemical and biophysical profiling, in addition to HDX-MS analysis of the monomeric protein itself (143). Lyophilization dramatically impacted the structural integrity of the latter and needs to be considered during experimental design and any estimation of aggregation kinetics. Moreover, the work further demonstrated the structural and functional importance of the C-terminal region of the protein.

#### Leucine-Rich Repeat Kinase 2

A second target of PD that has garnered significant attention in the development of novel neurotherapeutics is the large, multidomain Roco protein leucine-rich repeat kinase 2 (LRRK2), which as the name suggests, contains a signature leucine-rich repeat domain (144). The Roco family of proteins all have inherent Ras-like GTPase activity, and their regulatory roles are diverse and plentiful (145). Missense mutations in the LRRK2 gene results in increased susceptibility to both familial and sporadic PD, and is one of the strongest genetic risk factors for the latter (146, 147, 148). The G2019S mutation, for example, is widely found in Caucasian populations and has a prevalence of up to 40% in PD patients of Ashkenazi Jew and North African descent (149, 150). These mutations principally occur in the catalytic Ras of complex (Roc)-COR and kinase domains and thus likely drive PD pathogenesis *via* enhanced kinase activity and hyperphosphorylation of substrate proteins (151). Much effort has therefore been put into developing inhibitors of LRRK2 kinase activity (152). The full-length LRRK2 protein contains several functional and protein interaction domains (151). It is unusual in the sense that it contains both serine-threonine kinase activity, in addition to GTPase activity, which is under the control of its Roc domain. LRRK2 is primarily located in the cytoplasm but can also be found on the mitochondrial outer membrane (153). As such, the protein has been implicated in neurodegeneration through several pathways including autophagy, mitophagy, and calcium homeostasis (154).

In a first example, HDX-MS was used to decipher the conformational dynamics of GTP and GDP cycling in the regulation of the Roco protein of *Chlorobium tepidum* (155). By mapping to a crystal structure of the nucleotide-free LRR–Roc–COR domains, the authors were able to use the information derived from the HDX-MS to propose a pathway linking nucleotide binding to monomerization. The Taylor lab at the University of California have also used HDX-MS across several studies of LRRK2, more specifically, using a recombinant protein construct “LRRK2_RCKW_” which lacks the inhibitory N-terminus of the protein, thus maintaining full catalytic activity in the C-terminal half (156, 157, 158). As the kinase domain has been proposed as the primary activation site of the protein, the conformational dynamics of complex formation upon kinase inhibitor binding was therefore initially assessed (156). In the presence of the type I kinase inhibitor **MLi-2**, an overall reduction in solvent accessibility was observed throughout the protein, in agreement with a predicted compact domain architecture (159). By integrating the HDX data with molecular dynamics simulations, the authors were able to identify the LRRK2 kinase domain as an allosteric hub for its activation, in addition to characterizing significant long-distance crosstalk between LRRK2 kinase and GTPase domains. These observations were confirmed in a follow-up HDX-MS study on LRRK2_RCKW_ and a full-length inactive LRRK2 (fl-LRRK2INACT) monomer, identifying several regions which oversee conformational rearrangement in the protein, including the α3ROC helix, the switch II motif of the Roc domain, and the LRR–ROC linker (157). As these regions partake in significant interdomain crosstalk and allosterically impact both kinase and GTPase activities in LRRK2, they represent novel therapeutic targets of the protein. HDX-MS structural dynamics and mechanistic insights were confirmed by Gaussian accelerated molecular dynamic simulations, in addition to mapping to the cryo-EM structure of the protein (158, 160).

#### Ubiquitin Carboxyl-Terminal Hydrolase L1

Ubiquitin carboxyl-terminal hydrolase L1 (UCH-L1) is a member of the ubiquitin–proteasome system, where it acts as a DUB enzyme (161). It is one of the most highly expressed DUBs in the brain and central nervous system (CNS), with estimates suggesting that it comprises up to 5% of total soluble brain protein (162). The genetic association between UCH-L1 and neurodegeneration is subject to much debate, with one study identifying a point mutation in the *UCHL1* gene as a contributing factor to PD, while a polymorphism at S18Y confers a reduced susceptibility to the condition (163, 164). Furthermore, UCHL1 mutations have also been associated with ALS (165, 166). Such mutations can impact UCH-L1 catalytic activity and its aggregation into high-order species, and therefore, are a prime target for therapeutic development in PD and ALS. Structurally, UCH-L1 is characterized by a complex Gordian knot conformation and the link between the molecular arrangement of the protein and PD is still unclear. HDX-MS on a N-terminally truncated version of the protein, NT-UCH-L1, identified in murine brain tissue and confirmed in SH-SY5Y neuroblastoma cells, showed that this construct is more flexible and prone to aggregation compared to full-length, WT UCH-L1 (167). NT-UCH-L1 stability was influenced by monoubiquitination events and disulfide crosslinking and showed protection against reactive oxygen species. HDX-MS has also been used to characterize the structural destabilization of UCH-L1 by H_2_O_2_-mediated oxidation (168).

#### PD Protein 7

Finally, DJ1, also known as PD protein 7, is a deglycase which has been proposed to be neuroprotective by sensing and allowing cells to respond to reactive oxygen species and periods of oxidative stress (169). As such, it is essential for the maintenance of healthy mitochondria. The protein can inhibit the aggregation of alpha-synuclein, and mutations within its PARK7 gene result in an increased susceptibility to PD, making it an obvious candidate for the development of PD therapeutics (170). A recent study utilized HDX-MS to decipher the mechanistics of enhanced enzyme activity through the interaction of dimeric DJ1 with two separate activating peptides, ^15^EEMETIIPVDVMRRA^29^ and ^47^SRDVVICPDA^56^ (171). The dynamic HDX-MS information revealed unique peptide binding mechanisms to the α/β hydrolase core, facilitating improved substrate binding and enhanced enzyme activity.

### Alzheimer’s Disease

AD is the most common form of dementia, which refers to a family of conditions which display a progressive decline in memory, cognition, and language ability (172). AD pathogenesis is complex and caused by the concerted action of several factors including age, lifestyle, and environment. The disease also has a genetic component, with the ApoeE4 allele being the most common genetic risk factor for the condition. AD manifests sporadically, usually affecting those over the age of 65 (173). Molecularly-speaking, AD is principally characterized by the accumulation and deposition of toxic, fibrillar versions of two proteins, extracellular Aβ, and intracellular microtubule-associated protein tau (174). The aggregation and deposition of these two proteins in AD disrupt normal synaptic functioning, ultimately resulting in neuronal degeneration and dementia. As such, they are the prime targets for the development of novel therapeutic modalities against the condition (16).

#### Amyloid-Beta

Aβ is the principal component of the amyloid plaques that are found in the AD brain and has long been considered one of the key drivers of AD pathogenesis and progression. The protein is a hot target of AD therapeutics (174). Aβ is a normal product of metabolism, stemming from cleavage of the much larger transmembrane amyloid precursor protein by the concerted action of β- and γ-secretase enzymes (175). This results in a 37 to 49 amino acid residue peptide that is further cleaved to produce the predominant Aβ forms, Aβ40 (40 amino acids in length) and Aβ42 (42 amino acids in length) (176). It is still unclear which Aβ species represents its true pathological form, and as such, no fully effective therapies targeting the protein currently exist. Aβ monomers can assemble and indeed dissemble into higher order oligomers and protofibrils, and finally into large and insoluble amyloid fibrils, which have inherent neurotoxicity and form the basis of amyloid plaques (177). The oligomeric form is soluble and can be trafficked through the brain (174).

Aβ is an intrinsically disordered protein, and it has therefore been extremely difficult to generate meaningful structural data by traditional methods such X-ray crystallography. As HDX-MS is not limited by crystallization trials, it may represent an excellent alternative to assess the structural and conformational dynamics of the protein. As is the case with alpha-synuclein, several studies have used the unique information provided by HDX-MS to assess Aβ aggregation kinetics and inhibitors thereof. This was kickstarted with the work of Wetzel and colleagues who used a continuous labeling approach to monitor the conformational dynamics of different oligomeric Aβ40 species (178, 179, 180). The group identified a solvent protected and structured region at the center of the protein spanning residues 20 to 34 which likely initiates fibril formation and later used HDX-MS to demonstrate that calmidazolium chloride can trap and stabilize the protein in a protofibril-like conformation (181). Other studies using millisecond-continuous HDX-MS to monitor oligomer formation of Aβ40 showed that the rapid transition from monomeric to oligomeric species was underpinned by EX1-like exchange behavior encompassing two populations, one consisting of monomers and a second a heterogenous mix of dimers and tetramers (182).

The organization of Aβ40 and Aβ42 into a protected core capable of initiating aggregation and flanked by disordered and flexible N- and C-terminal regions has since been verified by others (183, 184, 185, 186) and Aβ42 has been shown to be more aggregation prone than another variant, Aβ43 (187). Recently, however, a functional role has been proposed for the N-terminal region of the protein, which has oligomer-stabilizing propensity, as demonstrated by gas-phase ion mobility separation and HDX-MS (188). The authors propose that the p3 peptide sequence covering residues 1 to 16 is required for the transition from small antiparallel oligomers to neurotoxic mature parallel cross-β structures.

In a very recent study, HDX-MS was used to determine the effect of heparin tetrasaccharides, dual promoters and inhibitors of aggregation, on Aβ (189). The technique monitored the formation of the glycosaminoglycan–peptide complex, which is primarily driven by its sulfation pattern. The region covering *V12HHQKL17* was identified as the primary interaction interface of glycosaminoglycan and Aβ, which was confirmed by molecular docking simulations. The authors also used a 3- (4,5-dimethylthiazol-2-yl)-2,5-diphenyltetrazolium bromide assay to demonstrate the anti-amyloid tendency of the heparin molecule and its therapeutic promise as an Aβ aggregation inhibitor (189). Sulphated oligosaccharides are a potentially cost-effect means of mopping up Aβ fibrils, with the added advantage of being able to easily traverse the blood-brain barrier.

Several mutations within the Aβ sequence of amyloid precursor protein can result in hereditary or familial AD, including the Arctic (E22G), Dutch (E22Q), English (D6R), Flemish (A21G), Iowa (D23N), Italian (E22K), and Japanese (D7N) variants (186). Illes-Toth and co-workers used a pulsed labeling HDX-MS approach to decipher the mechanistic impact that these mutations may have on Aβ42 aggregation kinetics. The results gave a bimodal pattern of two structural intermediates along the aggregation pathway, one solvent-protected (covering the central and C-terminal region) and one solvent-exposed (at the N-terminal half of the protein). The technique excelled at deciphering the different aggregation propensities introduced by the Aβ mutations, allowing a spectrum of aggregation kinetics to be resolved where the Arctic (E22G), Iowa (D23N), and Italian (E22K) mutants aggregate rapidly, the N-terminal mutations in the Japanese (D7N) and English (H6R) variants aggregating at a slower pace, with the Flemish (A21G) mutant slowest of all (186). A second study investigated the effect of the highly pathological Austrian mutation (T43I) on Aβ aggregation and kinetics (190), showing that this variant influences the transmembrane helix “hinge” region which is essential to its dimerization and cleavage.

Natàlia Carulla and colleagues have used the dynamic information provided by pulsed labeling HDX-MS to show that Aβ fibrils have a high degree of structural plasticity, constantly recycling and reforming between short-lived monomeric and multimeric states (177). Structural information on transient Aβ intermediates could pave the way for a whole new generation of aggregation inhibitors in AD and similar neurodegenerative disorders (19). The authors later confirmed the highly plastic and multistate nature of Aβ aggregation with follow-up pulsed labeling HDX-MS experiments, identifying distinct monomer, oligomer, and fibril populations of Aβ, the presence of which correlated with toxicity and aggregate formation (191). Notably, a higher degree of neurotoxicity was observed in the presence of increasing oligomeric species, which was in line with earlier reports from Qi and colleagues (192). The group has also shown that Aβ40 has a higher rate of fibril dissociation than its Aβ42 counterpart (193). As with alpha-synuclein, pulsed labeling HDX-MS has been used to help decipher differential aggregation kinetics of Aβ40 and Aβ42 (194), with the latter form showing increasing solvent protection over time, indicative of oligomer and aggregate formation. No change in HDX-MS aggregation kinetics was observed for Aβ40 over 48 h. Once again, due to having the highest solvent protection, the central section of Aβ42 was proposed as the key to the inherent aggregation propensity.

#### Microtubule-Associated Protein Tau

Microtubule-associated protein tau is another key player in AD. The protein provides stability and support to microtubules, aiding in their assembly, and promoting axonal outgrowth (37). One of the primary hallmarks of AD and similar tauopathies is the pathophysiological deposition of hyperphosphorylated, aggregated tau in the brain, resulting in aberrant synaptic communication and neuronal loss. Human tau exists as six different isoforms between 352 to 441 amino acids, generated by alternative splicing of the *MAPT* gene (195). Each isoform contains a total of zero, one, or two inserts (0N, 1N, and 2N) at their N-terminal end and either three or four carboxy-terminal repeat domains (3R or 4R). The native protein is soluble, intrinsically disordered and has little propensity to aggregate. Assembly of the protein into aggregated forms including paired helical filaments and neurofibrillary tangles underlies its toxicity (37). The protein structure itself can be broken down into several individual domains; the amino-terminal projection domain and the carboxy-terminal assembly domain, the latter containing a dedicated microtubule-binding region and aggregation. Both regions are connected by a proline-rich region containing several PXXP motifs which confer signaling properties (196).

Several groups have applied HDX-MS to study the assembly and aggregation of both monomer and fibrillar versions of tau (76, 197, 198). Many of the steps along the fibrillization pathway, that is, the structural transition from monomeric to oligomeric species of tau, still need to be elucidated. HDX-MS has been used to monitor conformational dynamics and solvent accessibility of the heparin-induced fibrillization of tau at various stages of aggregate formation, highlighting the stabilization of the R3 insert from the microtubule-binding region as a key player in the aggregation process (76). Both N- and C-terminal regions of the full-length protein (2N4R tau) remained largely solvent exposed following fibrillization. Results were confirmed by HDX-MS epitope mapping of commercially available tau antibodies.

Derek Wilson’s group at York University in Canada has pioneered the development of time-resolved electrospray ionization coupled to HDX to measure the conformational dynamics behind the hyperphosphorylation of tau on the millisecond timescale, confirming that the modification results in extended structure of tau, compared to the native protein, which may enhance amyloidogenic propensity (197, 198). The proline-rich region of tau can be heavily phosphorylated, and as such, a particularly recent study has used HDX-MS to assess the impact of phosphomimic mutations at T181, T217, T231, and S235 of this region on tubulin dimer binding and dynamics (196). Impaired tubulin binding was observed as a consequence of each mutation, with a knock-on reduction in microtubule polymerization. The results uncover the previously unknown importance of the proline-rich region in tau and tubulin interactions.

#### Apolipoprotein E

Apolipoprotein E (ApoE) is involved in the metabolism of triglycerides, cholesterol, and fat-soluble vitamins in the body and primarily produced by glial cells in the CNS (199). Three common isoforms of the protein exist, ApoE2, ApoE3, and ApoE4, with the presence of APOE∗ε4 allele being the most prevalent genetic risk factor for late-onset AD (173). Conversely, APOE∗ε2 is protective and APOE∗ε3 is neutral. All three isoforms can self-associate into oligomeric species. The protein itself is 299 residues in length and is comprised of several helices of an amphipathic nature. Two domains, each encompassing the N- and C-terminal halves of the protein, are connected through a flexible hinge region (200). From a functional perspective, the N-terminal region is more important through its ability to bind the low-density lipoprotein receptor. The C-terminal half on the other hand is involved in lipid binding. Researchers have utilized HDX-MS to monitor lipid binding to ApoE, its oligomerization states, and inhibitors thereof (201, 202, 203). In a 2011 study, the aggregation of WT ApoE monomers was compared to constructs harboring C-terminal mutations within residues 230 to 270 of the full-length protein, which may impact its self-association into higher order species (201). The contribution of this region to the oligomerization process was confirmed by differential HDX-MS kinetics between monomeric and oligomeric species. In a more recent study, HDX-MS was combined with numerical simulations to assess the structural heterogeneity of conformers and intermediates across intact apoE3 and apoE4 proteins (203). The authors simulated mixed EX1/EX2 kinetic behaviors as a representation of conformational heterogeneity and compared experimental HDX-MS data on several ApoE variants under native conditions. The ApoE4 isoform was shown to be less stable than its E3 counterpart, with a greater degree of conformational heterogeneity. As only a single mutation at C112R differentiates pathological ApoE4 and nonpathological ApoE3, the authors propose this to be a target for the screening and development of novel molecules that may be able to neutralize the apoE4 protein and force its structure into a more apoE3-like state (203). Expanding on this, HDX-MS was recently and elegantly combined with biophysical and molecular dynamic simulations to unravel the molecular mechanism of ApoE4 aggregation (204). Differences in HDX behaviors were confined to the N-terminal region surrounding position 112, including at the two helices covering residues 79 to 123 and 134 to 136. Interestingly, these regions were shielded from protease digestion in the ApoE homotetrameric form. Furthermore, differences in the HDX-MS behaviors of C112R mutant proteins were also observed the C-terminal domain of ApoE, suggesting that this region may be structurally important to the entire molecule and act as a self-assembly interface during its oligomerization (204). Finally, the authors performed *in vitro* aggregation and differential HDX-MS experiments to assess the effects of the drug candidate tramiprosate and the endogenous brain metabolite 3-sulfopropanoic acid on ApoE aggregation, both of which have demonstrated positive outcomes in AD patients with the APOE∗ε4 genotype (205). Both compounds appear to drive ApoE4 conformers to an ApoE3-like state. In a 2025 study, HDX-MS was used to compare the solvent accessibility of N-terminal and C-terminal domain interactions, for both apoE3 and apoE4 (206). For apoE4, the C-terminal domain influenced its N-terminal counterpart allosterically, resulting in a more open and solvent-accessible conformation, which was only partially mirrored in apoE3. This structural destabilization of the N-terminal domain in apoE4 may contribute to its pathological function. Finally, the authors suggest a high-throughput HDX-MS workflow could be used to screen molecules with “structure corrector” abilities targeting apoE4 destabilization (206).

#### Phospholipase C

HDX-MS has also been applied to structurally characterize several other proteins relevant to AD, including phospholipase C (PLC) enzymes (207, 208, 209). Dysregulation of this enzyme family has been implicated across various diseases through their action as key intracellular signaling molecules and diacylglycerol and inositol 1,4,5-trisphosphate second messenger generation (210). Strong genetic evidence has highlighted mutations in the PLCγ subfamily to be functionally important in AD (211). HDX-MS was combined with cryo-EM and crosslinking MS to assess the impact of rare variants on intact PLCγ1 enzyme structure and provide a mechanistic link to its dysfunction in disease (207). A detailed examination of the autoinhibited form of the protein, and its interaction with the intracellular sequence of fibroblast growth factor receptor 1 was provided. HDX-MS confirmed that the main binding and autoinhibitory interface of the two proteins is located within the nSH2 domain of PLCγ1. Increased PLCγ1 activity through a greater accessibility of the active site, similar to that observed in rare variants of the protein, further highlights the functional importance of this autoinhibitory interface. HDX-MS has additionally been used to determine the underlying mechanism and allosteric regulation of the enzyme in the presence of physiologically relevant lipid membranes (208).

#### Src Homology 2 Domain–Containing Inositol Polyphosphate 5-Phosphatase 1

A second signaling molecule which has been shown to be dysregulated in AD pathogenesis and therefore a key drug target is Src homology 2 domain–containing inositol polyphosphate 5-phosphatase 1 (SHIP1) (212). The protein may contribute to neuroinflammatory processes in the AD brain through TREM2 signaling and cytokine release in activated microglia (213). HDX-MS was integrated with X-ray crystallography and activity assays to decipher the structural architecture of the apo-, magnesium-, and phosphate-bound versions of SHIP1 and SHIP2, with and without its C2 domains, key drivers of phosphatase domain modulation (214). C2 plays a greater role in modulation in SHIP2 than SHIP1. The results can be harnessed for the generation of selective probes targeting the allosteric mechanism of SHIP enzyme activity.

#### Glial Fibrillary Acidic Protein

Glial fibrillary acidic protein (GFAP) is a type-III intermediate filament protein that is predominantly expressed on astrocytes and is involved in cytoskeletal organization, neuronal plasticity, and maintenance of blood-brain barrier integrity (215). The protein has been proposed as a biomarker for several CNS disorders with a reactive astrogliosis phenotype, including AD and PD (216). HDX-MS was thus used in combination with intact protein analysis and AlphaFold modeling to compare the structure and conformational dynamics of GFAP in the presence and absence of an artificial cerebrospinal fluid solution (215). The results showed that recombinant GFAP existed as a mix of several conformers and the addition of artificial cerebrospinal fluid lead to a more solvent-exposed conformation. Moreover, the protein contained several regions displaying EX1-like behavior, indicative of protein aggregation hotspots.

### Amyotrophic Lateral Sclerosis

ALS, which is also commonly referred to as motor neuron or “MND” is a relatively rare, progressive, and fatal disorder which leads to neurodegeneration of upper and lower motor neurons which oversee voluntary muscle contraction, eventually leading to muscle weakness and wasting (217). As of 2025, there are three drugs available to treat ALS, *riluzole*, *radicava*, and *tofersen*, with only marginal or conflicting benefits observed (218, 219). The primary disease-associated protein in ALS is TDP-43 (220). The protein is 414 residues in length and consists of an N-terminal domain which plays a role in protein oligomerization, two central RNA-recognition motifs (RRM1 and RRM2) for RNA binding, and finally, the C-terminal domain which is enriched in glycine residues and is the site of most PPIs for the protein. This latter domain also encompasses several residues which can be mutated in familial disease (221).

TDP-43 aggregation can be prevented by RNA binding, and HDX-MS was therefore recently employed to provide a detailed picture of the structural interplay of between RNA and TDP-43 complexes (222). Confirming the importance of RNA-recognition motifs 1 and 2 to RNA binding, a reduction in solvent accessibility was observed in these regions of TDP-43 upon RNA oligonucleotide binding. Furthermore, the authors highlighted differential HDX-MS behavior in a short α-helical region of the protein which may also be consequential to TDP-43 aggregation. In a second study, HDX-MS was employed to look at the contribution of mutation in the actin binding protein profilin-1 which may contribute to ALS pathogenesis (223, 224). The mutational switch of a glycine with a valine at position 117 of the protein may prevent actin polymerization and binding, enhancing ALS toxicity. Ghassempour and colleagues integrated HDX-MALDI-TOF MS and molecular docking simulations to determine the structural impact that this mutation has on profilin-1, revealing that the mutation enhances its fibrillization and aggregation.

### Spinocerebellar Ataxia Type 1

Spinocerebellar ataxia type 1 (SCA1) is a rare neurodegenerative disorder, primarily a result of an extended CAG repeat sequence in exon 8 of the ATXN1 gene, which encodes the ataxin-1 protein (225). SCA1 is an inherited, autosomal dominant condition, leading to a progressive decline in motor function, gait, and balance (226). No disease-modifying treatments are currently available for SCA1, but several therapies are in development, primarily targeting ataxin-1 (225). HDX-MS was elegantly employed to assess the structural interaction of ataxin-1 with 14-3-3 adaptor proteins, which indirectly contribute to ataxin-1 stability (227). Attention was focused on the ataxin-1 AXH domain for the development of molecules that prevent 14-3-3 interaction (228). The C-terminal half of ataxin-1 encompassing its AXN domain was confirmed to be intrinsically disordered in nature and this feature, including a phosphorylation event at Ser776, mediates its interaction with 14-3-3 (227). HDX-MS pinpointed a region of ataxin-1 spanning L709-E728 which likely interacts with 14-3-3 or causes a conformation rearrangement or shielding of these residues, which ultimately prevents complex formation. The authors postulate that this interaction has a chaperone effect on ataxin-1, reducing its dimerization, aggregation, and pathogenesis (227).

## Closing Remarks

It is an exciting time for the field of neurotherapeutic interventions for neurodegenerative disease. The recent, albeit moderate, success of mAb therapies for AD has provided a much-needed boost and optimism that similar strategies can be developed for PD, ALS, and others (87, 88, 89, 90). Indeed, several groups are working solidly on this endeavor (229, 230, 231, 232, 233, 234). In this review, we have focused on HDX studies which have incorporated bottom-up MS as the primary measurement tool. There are similar studies which have used analogous detection methods in combination with HDX to better describe neurodegenerative disease, including native MS, differential mobility spectrometry, and NMR spectroscopy (235, 236, 237). This highlights the versatility of the approach and also its potential to be integrated with other structural proteomics methods to encapsulate a more complete picture of neurodegenerative systems. The unique information on protein structural dynamics that HDX-MS provides is at its most powerful when successfully combined with approaches such as X-ray crystallography, cryo-EM and molecular dynamics simulations and there are numerous studies and reviews showcasing this (63, 236, 238, 239, 240, 241, 242). With the advent of more powerful MS instrumentation, higher throughput sampling robotics, the development of methods which can isolate HDX behaviors to within single amino acid resolution, and bioinformatic packages to handle large datasets, places HDX-MS as an essential tool in modern drug discovery and design workflows. As demonstrated herein, HDX-MS has provided unique and crucial structural insights into mechanisms of neurodegenerative disease, as well as laying out the framework for the development of novel therapeutics to tackle human disease.

## Conflict of Interest

The authors declare no competing interests.
